# Towards pump–probe single-crystal XFEL refinements for small-unit-cell systems

**DOI:** 10.1107/S2052252522011782

**Published:** 2023-01-01

**Authors:** Lise Joost Støckler, Lennard Krause, Bjarke Svane, Kasper Tolborg, Bo Richter, Seiya Takahashi, Tomoki Fujita, Hidetaka Kasai, Michihiro Sugahara, Ichiro Inoue, Eiji Nishibori, Bo Brummerstedt Iversen

**Affiliations:** a Center for Integrated Materials Research, Department of Chemistry, Aarhus University, Langelandsgade 140, Aarhus 8000, Denmark; b Department of Materials, Imperial College London, Exhibition Road, London SW7 2AZ, United Kingdom; c Department of Physics, Faculty of Pure and Applied Sciences and TREMS, University of Tsukuba, 1-1-1 Tennodai, Tsukuba, Ibaraki 305-8571, Japan; d RIKEN SPring-8 Center, 1-1-1 Kouto, Sayo-cho, Sayo-gun, Hyogo 679-5148, Japan; Harima Institute, Japan

**Keywords:** Serial femtosecond crystallography, XFEL, small-unit-cell structures, single-crystal structure refinement, data reduction

## Abstract

Serial femtosecond crystallography for small-unit-cell systems has so far seen very limited application despite obvious scientific possibilities. This is because reliable data reduction has not been available for these challenging systems. In particular, important intensity corrections such as the partiality correction critically rely on accurate determination of the crystal orientation, which is complicated by the low number of diffraction spots for small-unit-cell crystals. A data reduction pipeline capable of fully automated handling of all steps of data reduction from spot harvesting to merged structure factors has been developed. The pipeline utilizes sparse indexing based on known unit-cell parameters, seed-skewness integration, intensity corrections including an overlap-based combined Ewald sphere width and partiality correction, and a dynamically adjusted post-refinement routine. Using the pipeline, data measured on the compound K_4_[Pt_2_(P_2_O_5_H_2_)_4_]·2H_2_O have been successfully reduced and used to solve the structure to an *R*_1_ factor of ∼9.1%. It is expected that the pipeline will open up the field of small-unit-cell serial femtosecond crystallography experiments and allow investigations into, for example, excited states and reaction intermediate chemistry.

## Abstract 2

Using K_4_[Pt_2_(P_2_O_5_H_2_)_4_]·2H_2_O as a test case, we present newly developed serial femtosecond crystallography data reduction software for small-unit-cell systems featuring sparse indexing, seed-skewness integration, an overlap-based combined Ewald sphere width and partiality correction, and a sophisticated post-refinement and merging procedure.

## 1. Introduction

In recent years, X-ray crystallography on macromolecular compounds has seen significant progress through X-ray free electron laser (XFEL) studies (Chapman *et al.*, 2011 ▸, 2014 ▸; Schlichting, 2015 ▸). This is, among other things, because the short pulse durations of serial femtosecond crystallography essentially remove all effects of beam damage and atomic motion, and because the extreme brilliance reduces the crystallite size required for sufficient scattering intensity. However, in spite of the scientific and technological importance, progress for small-unit-cell systems has been very limited. A primary reason is the challenge of indexing reflections in serial crystallography frames, when the sampling of reciprocal space is limited, *i.e.* when the number of reflections on each frame is low. The cause is the relatively narrow spectral width of XFEL pulses produced by the self-amplified spontaneous emission (SASE) scheme and the lack of crystal movement during the femtosecond exposures, which limits the number of Bragg spots intersecting the Ewald sphere. This is contrary to orientation matrix determination for macromolecules, which is a highly overdetermined problem. A low number of recorded reflections is also a significant issue for hard-to-crystallize macromolecules such as, for example, membrane proteins, where small crystal size, disorder and noise effects limit the detection efficiency. This means that orientation matrix determination should be approached from a fundamentally different direction if serial femtosecond crystallography (SFX) is to be applied to small-unit-cell systems.

The initial step in any SFX data reduction is to apply various detector calibrations and identify the position of the diffraction peaks, commonly performed in a program such as *Cheetah* (Barty *et al.*, 2014 ▸). Any patterns found to contain significant diffraction peaks are passed to indexing programs such as *CrystFEL* (White *et al.*, 2012 ▸) or *cctbx.xfel* (Hattne *et al.*, 2014 ▸). For *CrystFEL*, the subprogram *indexamajig* auto-indexes and locally integrates the 2D peak intensities on each frame. The indexing is carried out by passing peak positions to robust auto-indexers developed for single-crystal X-ray diffraction such as *XDS* (Kabsch, 1993 ▸), *DirAx* (Duisenberg, 1992 ▸) or *MOSFLM* (Powell, 1999 ▸), or SFX-specific algorithms such as *Felix* (Beyerlein *et al.*, 2017 ▸) or *TakeTwo* (Ginn *et al.*, 2016 ▸). An auto-indexer commonly returns an orientation matrix (OM), *i.e.* a set of lattice vectors oriented in the laboratory frame. Most auto-indexers work by initially converting observed reflection positions into reciprocal lattice vectors and detecting periodicity in reciprocal space. In *MOSFLM*, for example, this detection is through projection of reciprocal lattice vectors onto a set of discrete directions. When a sufficient number of diffraction peaks contribute to the 1D projected histogram, sharp peaks are present in the Fourier transform of the histogram. In this case, the direction corresponds to one of the crystal principal axes, and the frequency provides access to the lattice parameter, allowing the OM to be determined. *Indexamajig* predicts peak positions on the original pattern based on this OM, and in the case of satisfactory agreement with detected peaks, integrates the peaks using simple box integration with corner background subtraction.

The resulting 2D-integrated squared structure factors are passed to a program capable of relative frame scaling and optionally also various post-refinement processes such as orientation matrix optimization and various corrections. The most significant correction, the partiality, accounts for the degree to which each particular lattice point overlaps with the Ewald sphere. If post-refinement of the OM is omitted or deemed unreliable, the partiality correction is often also skipped. Reasonable accuracy can, in many cases, still be achieved using much larger datasets (Kirian *et al.*, 2010 ▸; Li, 2016 ▸) as the stochastic variables such as crystal orientation (indirectly including partiality) and size variation average out between reflections. Finally, the optimized individually observed structure factors are merged.

The primary problem for small-unit-cell systems is that the number of diffraction spots on each frame is too low to allow reliable determination of 3D periodicity, meaning that determination of the OM and thus spot position prediction cannot be as straightforward as outlined above. In addition, common box integration is problematic for SFX data, in particular for nanocrystals, as the coherence of the XFEL beam and the finite size effects impact the diffraction spot shape and size significantly. Post-integration corrections and refinement are also contentious issues, where the community has not yet reached agreement on the optimal method. Some particularly pertinent issues are the simplifications of the important partiality and spectral width corrections and systematic intensity errors owing to the detection limit.

A recent contribution to the development of SFX on small-unit-cell systems was the successful structural determination of the small-molecule systems mithrene, thio­rene and tethrene (Schriber *et al.*, 2022 ▸). In this work, unit-cell parameters were obtained by accumulation of single-crystal diffraction frames into powder patterns, and the structures were solved in a continuous reindexing, rescaling and remerging process, using the partially obtained structure as a reference for later cycles (Schriber *et al.*, 2022 ▸).

We recently conducted experiments at BL3, SACLA, Japan (Tono *et al.*, 2013 ▸; Tanaka *et al.*, 2012 ▸) to identify and solve the primary challenges of small-unit-cell data reduction. Based on these measurements, we have developed extensive new software, incorporating and optimizing a range of both established and novel ideas to carry out all steps of the data reduction from raw SFX data to merged structure factors lists. The software is a robust pipeline featuring sparse indexing of multiple particles per frame, seed-skewness intensity integration and iterative orientation matrix post-refinement for reducing XFEL frames into merged structure factors for challenging systems with small unit cells. To test the accuracy of the data reduction process for small-unit-cell systems, the pipeline has been used to reduce data measured at SACLA on the small-unit-cell compound K_4_[Pt_2_(P_2_O_5_H_2_)_4_]·2H_2_O.

## 2. Experimental

### 2.1. Synthesis of K_4_[Pt_2_(P_2_O_5_H_2_)_4_]·2H_2_O

Single crystals of K_4_[Pt_2_(P_2_O_5_H_2_)_4_]·2H_2_O (PtPOP) were synthesized via a route inspired by Yamaguchi *et al.* (1990 ▸) and Che *et al.* (1985 ▸). 200 mg K_2_PtCl_4_ (Sigma–Aldrich, ≥99.9%) and 700 mg H_3_PO_3_ (Sigma–Aldrich, 99%) were dissolved in 1 ml of miliQ water. The beaker was covered with a petri dish to prevent water evaporation, and the solution was heated to 115°C in a sand bath and left overnight under continuous stirring. The following day, the solution was highly acidic, as expected from the reaction scheme (Roundhill *et al.*, 1989 ▸)

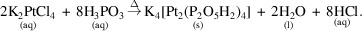

The solution was left to dry on the hot plate by removing the lid for ∼3–4 h. The solution did not dry out completely as the PtPOP crystals are sensitive to O_2_; thus, the solvent had to constitute a protecting layer. The product was washed with methanol and acetone to produce a yellow microcrystalline powder. The powder was dissolved in a minimum of miliQ water and recrystallized by slow vapor diffusion at ∼4°C using methanol as the more volatile solvent. After a week, the product was washed twice with methanol. Yellow, square-based cuboid-shaped crystals were obtained, exhibiting green phospho­rescence when illuminated with ultraviolet light, in accordance with the characteristic luminescent properties of PtPOP compounds (Pinto *et al.*, 1980 ▸).

### 2.2. Conventional X-ray diffraction on K_4_[Pt_2_(P_2_O_5_H_2_)_4_]·2H_2_O

**Figure 1:**
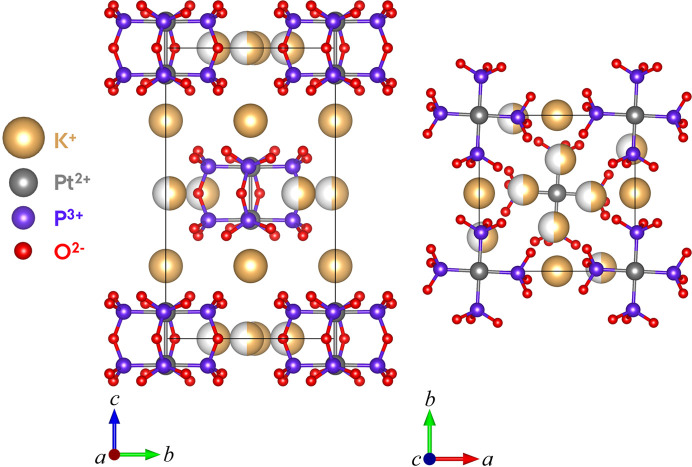
*I*4/*m* structure of K_4_[Pt_2_(P_2_O_5_H_2_)_4_]·2H_2_O. The K^+^ ions at the 8*h* sites (*z* = 0, 0.5) have an occupancy of 0.5. The water molecules and the hydrogen atoms have been omitted.

One of the synthesized PtPOP single crystals was cut into a cuboid with the dimensions ∼100 × 100 × 40 µm, and an X-ray diffraction experiment was carried out at an in-house single-crystal diffractometer (SuperNova). The diffractometer uses an Mo *K*α beam (λ = 0.71 Å), and the sample-to-detector distance was 53 mm. The initial data reduction was performed in *CrysAlis PRO* (RigakuOD, 2021 ▸), and the structure was subsequently solved in *Olex2* with *SHELXT* and refined with *SHELXL* (Sheldrick, 2015*b* ▸,*a* ▸; Dolomanov *et al.*, 2009 ▸), using a weighting scheme and anisotropic displacement parameters (ADPs). The synthesized PtPOP crystals were found to crystallize in the tetragonal space group *I*4/*m* with the unit-cell dimensions *a* = 9.3113 (3) Å and *c* = 15.9547 (11) Å. The exact location of the water molecules proved difficult to determine. Excluding the water molecules left a significant amount of unaccounted residual electron density in certain areas (Δρ_max_ ≃ 4.3 e Å^−3^). By inserting oxygens corresponding to the crystalline water molecules at the sites of the highest residual density (but still omitting all hydrogen atoms), the final structure had an *R*_1_ factor of ∼2.89%. However, we have not been able to unambiguously determine the exact positions of the oxygens of the water molecules, and in order to be able to compare the structural data with data measured at SACLA (presented in Section 6), we have excluded the water molecules and the hydrogen atoms in the [Pt_2_(P_2_O_5_H_2_)_4_]^4−^ units altogether. By doing so, we obtain an *R*_1_ value of 3.68%. Crystal data, data collection and structure refinement details are summarized in Table 1 ▸ for the crystal structure of K_4_[Pt_2_(P_2_O_5_H_2_)_4_]·2H_2_O with the water molecules and hydrogens omitted. The *I*4/*m* structure of K_4_[Pt_2_(P_2_O_5_H_2_)_4_]·2H_2_O is visualized in Fig. 1 ▸ without the hydrogen atoms and water molecules. The K^+^ ions occupy two different sites: 4*d* sites at *z* = 0.25 and 8*h* sites at *z* = 0, 0.5. The K^+^ ions at the 8*h* sites have an occupancy of 0.5, thus the charge balance is fulfilled.

### 2.3. XFEL data collection

At SACLA, we used a monochromatic X-ray beam with an X-ray energy of 17.00 keV (λ = 0.7293 Å) and an 8-module multi-port charge-coupled device (MPCCD) Octal Phase-III detector (Tono *et al.*, 2015 ▸; Kameshima *et al.*, 2014 ▸). The detector was offset by 35 mm to an upper position, covering an angular 2θ range from 5 to 55°. To optimize the signal-to-noise ratio (SNR) and therefore the data quality, the detector was moved close to the sample at a distance of 50 (2) mm, thereby increasing the number of Bragg spots being measured. The beam size was focused to approximately 1.1 × 3.4 µm using a Kirkpatric–Baez mirror system. The sample was dispersed in a grease matrix (Sugahara *et al.*, 2014 ▸) and extruded using a piston system to control the flow rate. A helium flow was used to stabilize the grease extrusion, and the flow rate was 0.02–0.50 ml min^−1^. The nozzle size was varied between 100 and 200 µm depending on the crystallite size distribution. The particle concentration in the grease proved difficult to control accurately, but we aimed for an average of one crystallite per shot. The average pulse energy was ∼80 µJ at the sample position, and the average wavelength spectrum was described well by a Gaussian function with a full width at half-maximum of 46.7 eV. No further monochromatization aside from the inherent SASE process was applied. The XFEL machine was operated at 30 Hz. A 1.0 mm Si attenuator was used for most samples to avoid excessive saturation of pixels on the detector. The exposure time of the detector was 1.0 ms, and the detector was synchronized to the XFEL machine to ensure that the diffraction images were taken in a pulse-by-pulse manner.

Detector calibration was carried out using ∼28.000 data frames measured on a relatively high concentration of CeO_2_ in the grease matrix (Sugahara *et al.*, 2014 ▸). The data frames were summed to yield powder-like rings, and the pattern was used for the initial calibration through the *pyFAI* algorithm (Prescher & Prakapenka, 2015 ▸). The calibration relies on using the position of powder rings of a known standard to refine the point of normal incidence, sample-to-detector distance and detector geometry (pitch, roll and yaw). The relatively low number of diffraction spots in each frame of a normal XFEL measurement may, in some cases, give prohibitively low powder ring intensity.

## 3. Data reduction pipeline

Though numerous data reduction routines for SFX data have been published in recent years, no adequate software for end-to-end processing of small-unit-cell SFX data has been published yet. We aim to solve that deficiency with a tailor-made pipeline capable of automatically processing data directly from raw data frames. The computationally expensive operations are parallelized and optimized to allow fast processing from collected frames to a corrected structure factor list.

**Figure 2:**
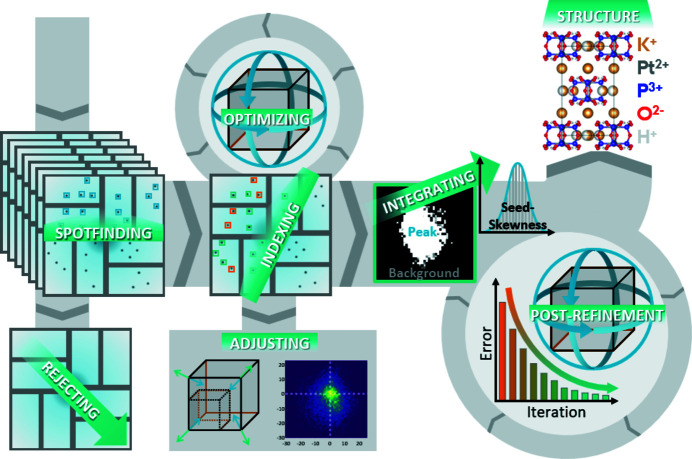
Schematic of the SFX data reduction pipeline including initial spot-finding, indexing using an optimized version of the SPIND algorithm, integration based on a seed-skewness method and a post-refinement process including various corrections. The final output is a list of merged structure factors.

Data reduction for SFX data can be broken down into a relatively simple sequence of spot-finding, indexing, integrating, correcting (optionally) and merging. The individual data processing steps of the pipeline are detailed in separate sections below, with an overview of the whole process shown in Fig. 2 ▸. Between each step, the output is saved to allow partial processing. The algorithm requires that the unit-cell lengths and the crystal space group are approximately known. However, this will not be an issue for most applications.

In order to test the pipeline, pump–probe measurements have been carried out using the synthesized single crystals of PtPOP [*a* = 9.3113 (3) Å, *c* = 15.9547 (11) Å] as our test sample. PtPOP has a special standing in the field of excited-state crystallography (Cole, 2008 ▸), and it has been used extensively in pump–probe experiments at different time-scales (Kim *et al.*, 2002 ▸; Christensen *et al.*, 2009 ▸; van der Veen *et al.*, 2009 ▸; Thiel *et al.*, 1993 ▸). Overall, 695 000 frames were measured with a hit rate of ∼7%. In the following, a data frame from the measured PtPOP dataset has been chosen to showcase the pipeline results.

### 3.1. Spot finding

After detector calibrations, the first step of the pipeline is to identify the positions of the diffraction peaks. Finding the position of intense Bragg spots on a relatively even background is a computationally expensive but quite simple process. To avoid falsely finding random noise, each frame is initially smoothed using a Gaussian filter. The image is then subjected to a dilation process where a structuring element – in this case, a plus-sign-shaped mask consisting of 5 pixels – is used to expand the high-intensity areas on the image by convolution. By placing the mask on the original image, the dilated image is created by assigning the maximum intensity inside the mask to the central pixel of the mask on the corresponding dilated image. Afterwards, local maxima closer than the dilation size are merged. This is done to avoid single spots being incorrectly identified as multiple spots because random detector noise has caused the spots to split into several, closely spaced, local maxima. Maxima are then located where the original image is equal to the dilated image and the intensity is more than 5 times the median of the smoothed frame. The process is sped up significantly by skipping any frames where the maximum pixel intensity is not more than 25 times the mean pixel intensity. This requires masking of all hot or dead pixels on the detector. Testing confirmed that this did not skip any frames that would otherwise have been included in the final merging process.

Detector coordinates of identified spots are translated into scattering vectors based on the refined detector geometry. Each spot is checked and tagged for pixel saturation, unreasonable mosaicity, weak maximum intensity and too-large scattering angle (2θ) deviation to the closest predicted scattering angle for the crystal. The spot intensity relative to the estimated uncertainty is determined through a seed-skewness integration as detailed later in Section 3.3. The tags serve to rank the spot trustworthiness for the indexing algorithm.

The MPCCD detector used for the SACLA experiments is subject to considerable noise, meaning that weak spots are not necessarily easily distinguishable from the background. This means that weak spots will not be found unless they are located close to the center of the Ewald sphere, giving systematic overestimation. We avoid this issue by using a refined spot-search-and-reject algorithm locally around predicted spot positions during the integration step, detailed in Section 3.3. As such, the initial spot finding is only important for an initial guess of the OM, and the spot finding limits are therefore deliberately set conservatively to avoid the inclusion of false spots. Unidentified spots will be included later if they agree with the predicted spot positions derived from the calculated orientation matrix.

**Figure 3:**
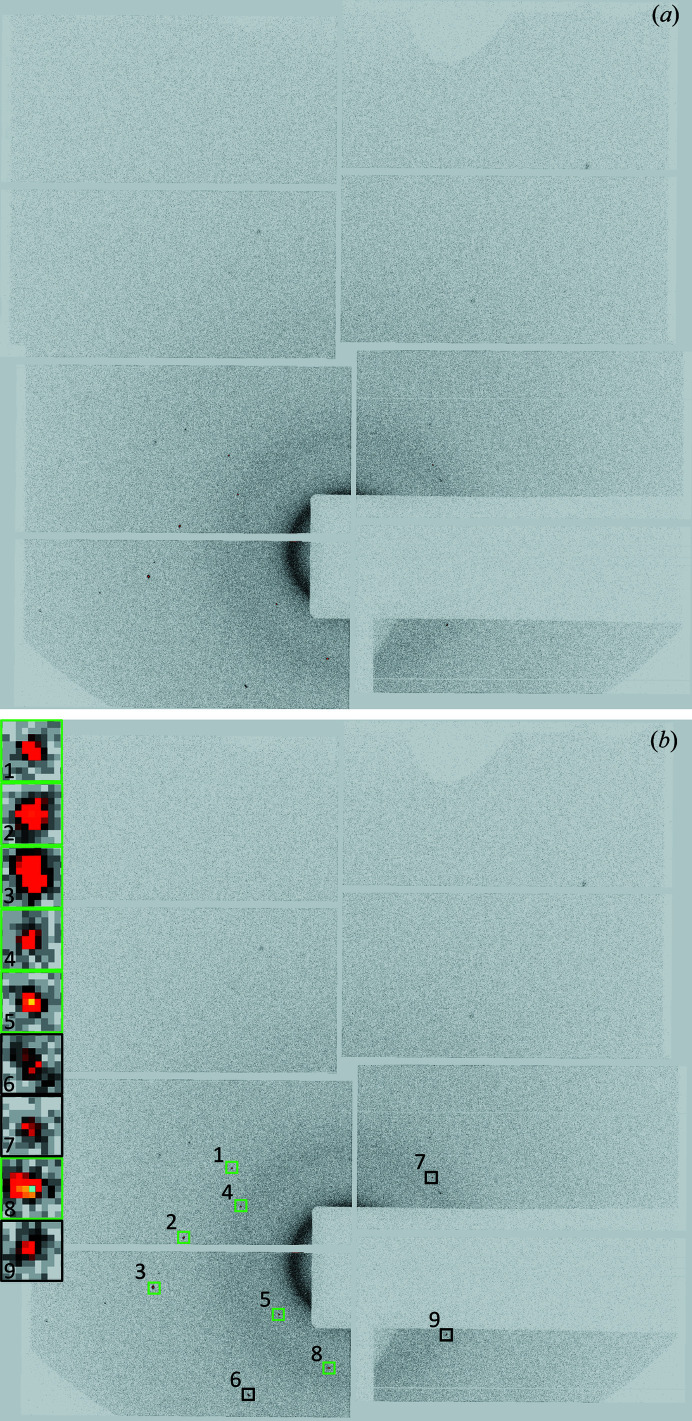
(*a*) Raw detector frame from the PtPOP dataset. This frame will be referred to as Data Frame 1. (*b*) During the spot finding process, most of the intense spots are found (indicated by the six green squares). Unidentified spots can be included later if they agree with the predicted spot positions derived from the calculated OM, which is the case for the three spots enclosed by black squares. Zoomed-in insets of the nine spots are shown on the left.

An example of a data frame where a PtPOP crystal has been hit is shown in Fig. 3 ▸(*a*). This data frame will be referred to as Data Frame 1 and will be used throughout the paper to exemplify the data reduction process. In Fig. 3 ▸(*b*), the spots detected on Data Frame 1 during the spot finding process are indicated by green squares.

### 3.2. Indexing

**Figure 4:**
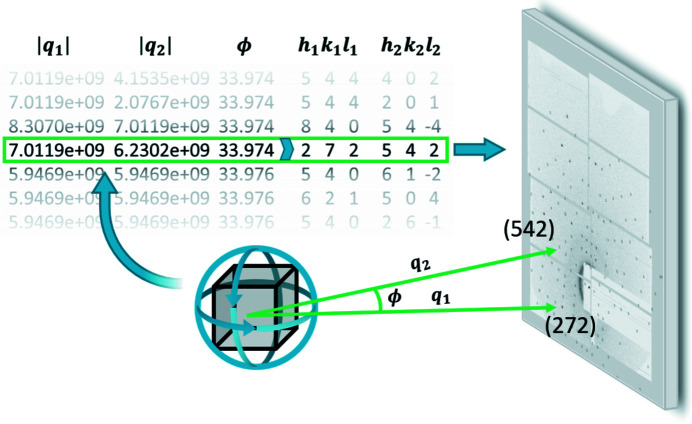
OM determination from a pair of reflections based on the SPIND algorithm. Scattering vector lengths and angles between them are calculated based on observed spot positions. On comparison with a reference table based on known unit-cell parameters, the reflections are indexed. The orientation matrix relative to the laboratory frame is then easily determined as the matrix that rotates the same reflections on top of each other.

Indexing is based on a heavily modified and optimized implementation of the sparse pattern indexing [SPIND (Li *et al.*, 2019 ▸)] algorithm. The algorithm proceeds as follows:

(1) Prior knowledge of unit-cell parameters and crystal symmetry is used to automatically generate a reference table containing the lengths and angles between all possible combinations of scattering vectors within a user-defined resolution limit.

(2) All spots on a given frame are ranked based on the flagging carried out during spot finding. Integrated intensity divided by the estimated standard deviation of the spots is used to order spots given equal rank. Spots below a user-defined minimum rank are rejected, which are typically only contaminant peaks (based on 2θ).

(3) If the number of remaining spots on a frame exceeds a user-defined number, the indexing begins. We found that a minimum of 5 spots per frame proved to be a reasonable compromise between the number of frames that would thereby be rejected and the resulting unit-cell accuracy. Scattering vector lengths and angles corresponding to each possible spot pair are compared with the entries of the reference table. For each match within a user-defined tolerance between an observed peak pair and the reference table, a corresponding crystal orientation is calculated and added to a list of potential solutions. This procedure therefore allows multiple crystallites to be indexed on the same frame. Spots not matching any other spot are replaced with the highest ranked excluded spot.

(4) For each potential OM, a new set of indices is calculated for scattering vectors of all the spots found. The solution is rejected if it predicts fewer than the user-defined number of spots (5) to have integer *hkl*s within a pre-set tolerance. If a solution is not rejected, it is refined to minimize the distance to all spots within the tolerance, iteratively including additional spots as they come within the tolerance. The minimization is done in the detector pixel coordinate system to equalize weighting between spots close to and far from the point of normal incidence on the detector. This choice means that the position of scattering vectors perpendicular to the detector plane is not refined in the interest of algorithmic speed. This is addressed later in the algorithm. Solutions are scored based on (1) the match rate and (2) the mean distance between the predicted and observed spot position for matched spots.

(5) The best solution is saved as the OM of a crystallite in the frame. Any spots indexed by this solution are removed from the pool of unindexed spot positions.

(6) For all indexed scattering vectors, the predicted spot position on the detector is calculated, assuming integer *hkl* values. If a peak has been detected within a short distance from this spot, the scattering vector is included in the subsequent OM refinement. The OM is refined by minimizing the summed distance from all predicted reciprocal lattice points (RLPs) to the Ewald sphere and to the corresponding detected spot using a suitable weighting scheme. This corresponds to maximizing the partiality of all spots.

(7) The procedure is repeated from (3) until the number of remaining unindexed spots is below a user-defined threshold, essentially resulting in an OM for each illuminated crystallite that gives rise to a sufficient number of detectable diffraction spots. The OMs are saved to be used to predict approximate spot positions and correction factors during the integration routine. They are updated during post-processing. A schematic of the indexing of a single spot pair is shown in Fig. 4 ▸.

**Figure 5:**
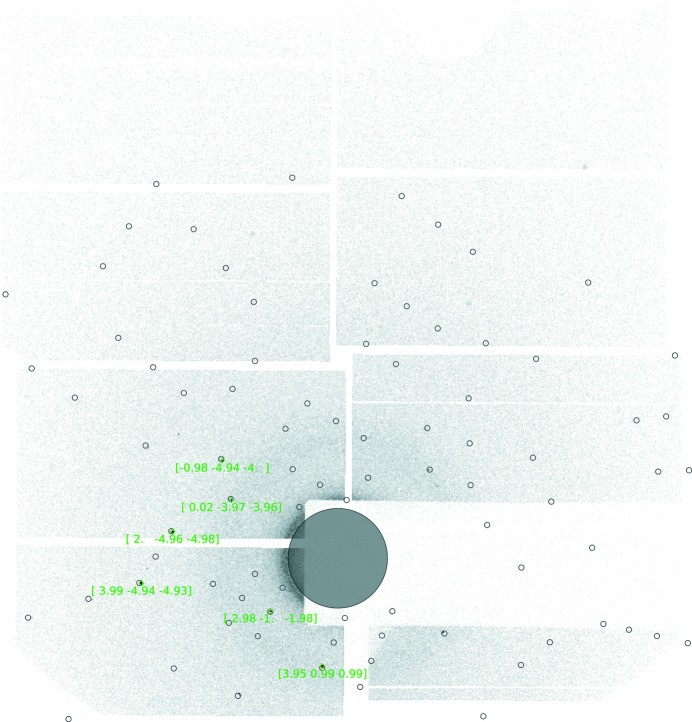
Indexing of the initially found spots on Data Frame 1. Diffraction spots from one crystal have been identified and the orientation matrix determined. Note that all assigned *hkl* values are close to integer numbers (the pre-set tolerance was 0.15). Rejected spots would be enclosed in red squares, but no spots have been rejected on Data Frame 1 during the indexing process.

Compared with the original SPIND algorithm, a reduction of reference table symmetry-redundancy and a computationally more efficient pre-sorting and search algorithm have resulted in a substantial speed increase. Contrary to SPIND, all potential solutions are optimized prior to selection of the best solution. This is made possible by a highly efficient OM refinement routine. In addition, the modified algorithm allows simultaneous indexing of spots belonging to different crystallites on the same frame. However, to avoid the ambiguity of erroneously indexing spots to more than one OM, the multiple OM indexing feature was not utilized in the data reduction of the PtPOP data, meaning that only one OM was allowed on each frame. In Fig. 5 ▸, Data Frame 1 is shown after the indexing process.

**Figure 6:**
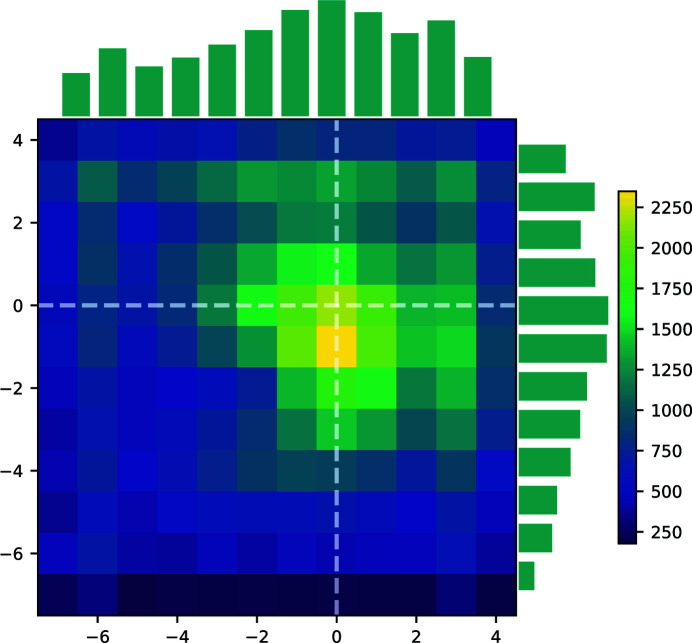
2D heat map of the predicted spot positions relative to the observed spots for ∼122.000 PtPOP diffraction spots. The units on the axes are in pixels. 1D histograms are shown above and to the right of the plot. (0, 0) has been indicated with white dashed lines. None of the differences in predicted and observed spot positions fall outside the limits of the heat map.

Although the algorithm is reliant on having a predetermined unit cell, it is well known that slight differences in unit-cell parameters are obtained between different experiments. This is either due to small changes in the crystal composition, accuracy of wavelength determination, uncertainty in the known unit-cell parameters or similar minor unavoidable issues. To prevent this effect unduly hindering the indexing of reflections, for each dataset, an initial overall optimization of the unit cell is performed based on a 2D histogram of differences between the predicted and observed diffraction spot positions for all frames collected (and successfully indexed) on that sample. The mean 2D spot position difference between predicted and observed spots should be zero for the correct average unit cell. This is used to adjust the known unit-cell parameters for all subsequent runs. A histogram showing the differences between the predicted and observed diffraction spot positions for the adjusted unit cell of PtPOP based on ∼122.000 diffraction spots is shown in Fig. 6 ▸. The plot indicates that the mean unit-cell parameters are reasonable as the maximum is near the center at (0, 0). Furthermore, as the distributions are relatively narrow, the predicted spot positions do not seem to be affected much by the small variations in the unit-cell lengths for each individual OM.

### 3.3. Integration

The basic premise of the integration routine is to predict RLPs close to intersecting the Ewald sphere, locally search the raw data frames at predicted peak positions, integrate any found peaks and correct the resulting intensities appropriately.

Using the optimized OMs obtained from indexing, all possible *hkl*s within the boundaries of the detector are determined and their corresponding scattering vectors are calculated. This means that the integration routine is not limited to the peaks detected during the initial spot finding. From the scattering vectors, diffracted beam vectors are calculated. To optimize the speed of computation, an initial rejection based on the length of the diffracted beam vector relative to the radius of the Ewald sphere is carried out. Scattering vectors predicted to fall outside the detector area or on the edge of any detector modules are rejected. Each remaining scattering vector is treated independently. The actual spot position is adjusted based on the maximum intensity within a small box around the predicted peak position.

An integration box around the found peak is constructed. Prior to the computationally expensive integration, the spots are subjected to a series of rejection tests. A spot is rejected if the maximum intensity is too low relative to the estimated frame noise. Frame noise is estimated using a simple and computationally inexpensive 2D convolution with negative nearest-neighbor and positive next-nearest-neighbor contributions (Immerkaer, 1996 ▸). Inspection of hundreds of integrated frames confirmed that a reasonable cut-off ratio was 20 times the frame noise (this strongly depends on the detector). The presence of a peak is further confirmed by testing that the intensity distribution within the integration box has a significant Fisher–Pearson coefficient of skewness. Peaks containing saturated pixels, which on a CCD detector leads to significant bleeding into adjacent pixels and unreliable intensity estimates, are detected if 5 or more pixels are within 1% of the maximum intensity of the box.

**Figure 7:**
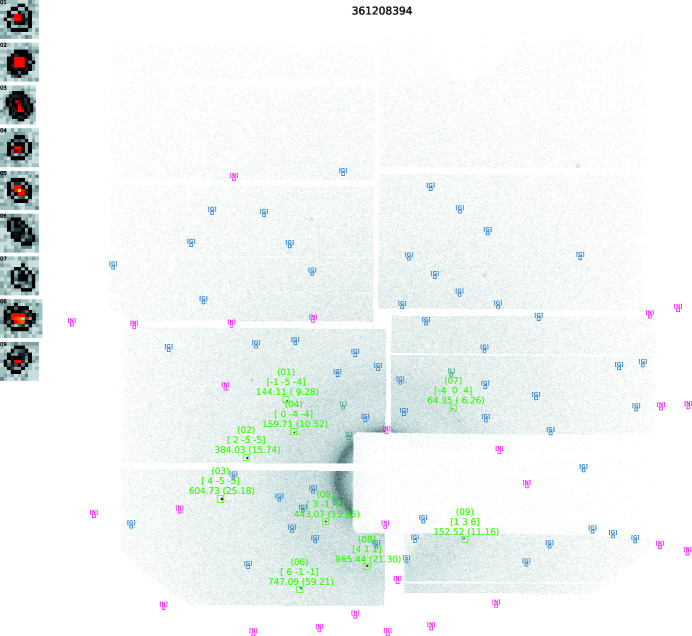
Data Frame 1 after the integration process. Reflection indices are given above the spot positions along with the calculated spot intensities. Nine spots have been integrated, and the integration boxes with the integrated spots are shown in the left corner. By comparing with Fig. 3 ▸, it becomes apparent that spots can be included in the integration even if they were not found during the initial spot finding process (spot numbers 06, 07 and 09). This is because all positions corresponding to symmetry-allowed reflections are searched for peaks.

The curated list of reflections is integrated using the seed-skewness method (Bolotovsky *et al.*, 1995 ▸; Bolotovsky & Coppens, 1997 ▸), which relies on dynamically growing the peak area from an initial pixel using the maximum adjacent intensity until the skewness of the intensity distribution within the integration box is lower than 3 times the variance of the intensity distribution. If the peak area reaches the edge of the integration box before this occurs, the peak is rejected. The integration box indirectly acts as the maximum allowed peak size and is adjusted globally by the user. After determining which pixels are assigned to the background and which contribute to the peak, any islands or background pixels surrounded by peak pixels are assigned to the peak. The intensity is obtained by summing the peak area, from which the background is subtracted based on the non-included pixels in the integration box. This active box grows with the peak area to ensure a reasonable ratio between the peak area and the background area. The seed-skewness method has the great advantage of providing accurate intensities despite high noise levels and weak reflection intensities in a statistically well founded manner. It also handles non-circular peaks particularly well, contrary to many other common integration routines, which is particularly important for nanocrystal investigations. The implementation has been heavily optimized for speed of integration, as the dynamic seed-growth is inherently a slow process. In Fig. 7 ▸, Data Frame 1 is displayed after the integration process with insets showing the obtained integrated spots.

## 4. Intensity corrections

Having obtained the integrated intensities, a host of corrections are applied. Integrated peak intensities are corrected for spectral spread (sometimes referred to as pseudo-Lorentz factor), partiality, polarization, angle of incidence, detector gain and incoming beam intensity. For example, the angle of incidence correction adjusts for the non-uniform quantum efficiency (QE) of the detector at different angles of incidence. The thickness of the MPCCD sensor (*d* = 300 µm) is insufficient to detect all photons impinging on the detector. Using the sensor thickness and the linear absorption coefficient, the QE can be calculated to be around 37% at an energy of 17 keV under normal incidence conditions.

Furthermore, there are some inherent biases in the seed-skewness integration which are corrected for using the corrections termed ‘hidden tails’ and ‘included noise’ (Bolotovsky & Coppens, 1997 ▸). Although not a direct intensity correction as such, the tendency to have a better chance of detecting peaks with RLPs centered on the average Ewald sphere is also handled using a detection level weighting prior to the post-refinement. Absorption corrections might also be important for systems like PtPOP. However, owing to the limiting sampling per shot, it is challenging to incorporate absorption corrections without also introducing errors. The solution to this problem is still being evaluated.

The intensities are corrected for partiality using a method similar to Uervirojnangkoorn *et al.* (2015 ▸), which is based on a derivation by Kahn *et al.* (1982 ▸). The incidence angle correction is based on the procedure described by Zaleski *et al.* (1998 ▸). The incoming beam intensity is measured in the optics hutch using a beam monitor. Correcting for the incoming beam intensity is primarily relevant for the subsequent rejection criteria, as the relative frame scale is refined in the post-refinement. Besides the polarization, the angle of incidence and the incoming beam intensity corrections, the remaining corrections are detailed below, as they differ from results published elsewhere.

### 4.1. Spectral width

**Figure 8:**
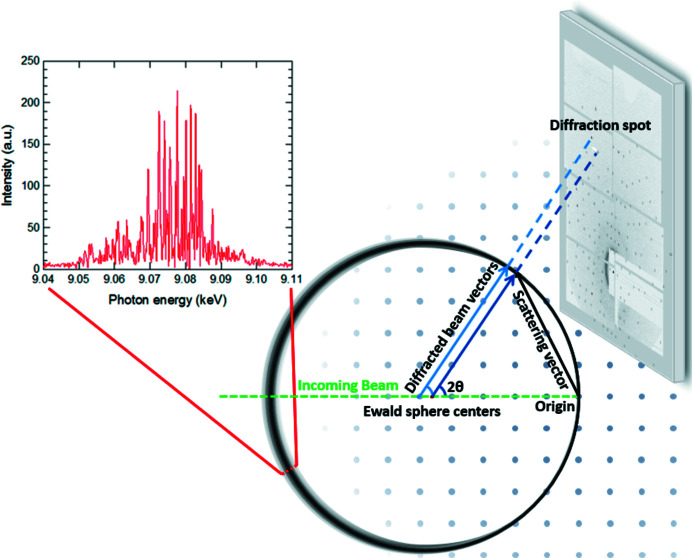
Schematic of the scattering geometry. Spectral width (black circle) and reciprocal lattice point size (gray spots), exaggerated for clarity. The inset shows an example of a measured spectral distribution from BL3 at SACLA when using a 9.07 keV beam (Inubushi *et al.*, 2017 ▸). Note that the photon energy used in our experiments was 17.00 keV.

The basic premise of a spectral width correction is that X-rays from a real source are not entirely monochromatic. This means that instead of having an infinitely thin Ewald sphere, we have a spheroid of a finite thickness. A coordinate system in which the center of the reciprocal lattice lies at (0, 0, 0) and where the *z* axis points along the beam can be defined. As the Ewald sphere intersects the origin of reciprocal space and has a radius of 1/λ, in this coordinate system, it is centered at (0, 0, −1/λ). This means that a spread in the wavelength of the incoming beam will give a range of Ewald sphere centers, which ultimately makes the average Ewald sphere slightly elliptical. The wavelength distribution also contributes to the finite width of the Ewald sphere. A schematic drawing of the situation is shown in Fig. 8 ▸.

The width increases from 0 at the origin of reciprocal space to 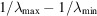 along the −*z* direction. As the same intensity is distributed over the thickness of the Ewald sphere regardless of direction, the spectral width correction, *S*_sw_, is proportional to the inverse of this width, *d*_Ewald_:

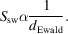

The time-averaged spectrum is well described by a Gaussian function, though individual pulse spectra are spiky, as can be seen from the inset in Fig. 8 ▸. The width can then be described in terms of the standard deviation of this distribution. The relative Ewald sphere thickness as a function of scattering angle and spectral width can, for small spectral widths to a very good approximation, be calculated from simple trigonometric considerations as

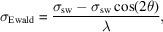

where σ_sw_ is the (known) standard deviation of a normal distribution fitted to the spectral distribution. For the beamline and wavelength used in our experiments, this was measured at the beamline using a method described by Inubushi *et al.* (2017 ▸). The spectral width correction has been included in the implementation of the partiality correction described below.

### 4.2. Partiality

A significant challenge in any SFX experiment is that there is no continuous rotation of a crystal, meaning that RLPs are not rotated through the Ewald sphere while data are being collected. Spots on collected frames instead represent instantaneous intersections between RLPs and the Ewald sphere. The intensity of a spot depends on the degree of overlap between the finite volume of the RLP and the finite thickness of the Ewald sphere. Each reflection can be assigned a value equal to the product of these two functions, termed the partiality, which can be used to correct the resulting intensity to the true RLP intensity. The partiality can thus conveniently be calculated as the product of the RLP density function and the Ewald sphere density function in reciprocal space. The Ewald sphere density along the diffracted beam vector is appropriately described by a normal distribution with the width 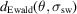 , which is centered on the position of the average Ewald sphere. The RLP density function arises from the fact that, for any real crystal, the RLP is in fact not a point. Finite crystallite size, mosaicity, crystal shape *etc.* spread the RLP over a volume in reciprocal space. However, the contributions affect the RLP in different directions. For example, variation in the *d* spacing of the reflection increases the size of the RLP perpendicular to the vector from the reflection towards the Ewald sphere center, while mosaicity will contribute to an increased size perpendicular to the vector pointing from the reflection towards the origin of reciprocal space. A strict derivation of the shape of the RLP density function relies on accurate determination of a prohibitively large number of crystal and setup parameters. Instead, we opt to assume that the function is reasonably described by a spherically symmetric 3D Gaussian centered on the calculated RLP position. The width can be determined by fitting a 2D Gaussian directly to the observed 2D intensity of each collected reflection. This implementation has been included in the code. However, this gave rise to an unreasonably large variation in the RLP width between peaks even when the peaks originated from the same crystallite. At present, the RLP width is set to a reasonable number based on manual inspection and fitting of well resolved peaks. This is not expected to be the final implementation, but rather a working approximation. The calculated position is based on the known OM and the assigned *hkl* of the reflection instead of the found position, as the RLP is not necessarily centered on the Ewald sphere.

**Figure 9:**
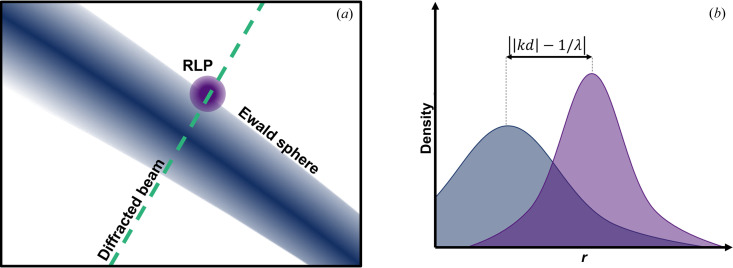
Schematic of the combined spectral width and partiality correction. (*a*) A cut-out of the Ewald sphere intersection with a single RLP is shown. (*b*) *S*_par_ is calculated from the integration of the product of the Ewald sphere density and the RLP density function along the diffracted beam path, *r*.

The combined partiality and Ewald sphere width correction, *S*_par_, then becomes a 3D spatial integral over the product of two normal distributions with known means and widths. As the Ewald sphere local density in the vicinity of a single RLP can be assumed to be flat perpendicular to the diffracted beam vector, and the RLP is assumed to be well described by a spherically symmetric Gaussian, this simplifies to a 1D integral along the diffracted beam vector, written as

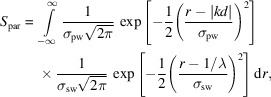

where *r* is the coordinate in the direction of the diffracted beam vector, which is the vector from the average center of the Ewald sphere towards the RLP. |*kd*| is the distance from the average center of the Ewald sphere to the calculated position of the RLP. σ_pw_ is either the average standard deviation of the 2D Gaussian fit to the collected peak or, in the case of uncertain peak fits, it can be set to a fixed value for all peaks as mentioned previously. The functions are individually normalized so each function integrates to one. A schematic illustration of the geometry in 2D and along the integration coordinate *r* is shown in Fig. 9 ▸.

The expression for *S*_par_ can be simplified, reducing the necessary computation time. The following definite integral is known (Spiegel *et al.*, 2013 ▸):

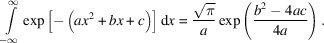

The terms of *S*_par_ are rearranged to

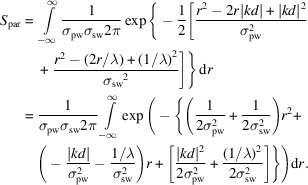

Applying the definite integral gives

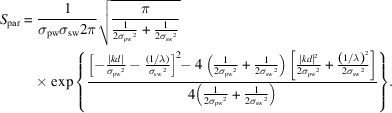

As the widths are real and positive, the expression can be simplified to

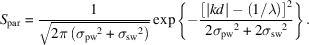

As the partiality is a relative measure between reflections, the numbers can be scaled. To obtain a number close to unity, the final expression for the combined spectral width and partiality correction is

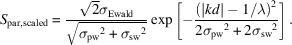

This correction method is based on the averaged pulse spectrum, as the spectrum of individual pulses varies due to the stochastic nature of SASE radiation and is not possible to measure for each. However, as the measured values are related to an integral of a smooth function (the RLP) that will average out peaks and dips in the spectrum quite efficiently, this correction will not systematically affect specific reflection families and is thus just an additional source of noise. We are not aware of a similarly complete partiality and spectral width correction that has been implemented elsewhere.

### 4.3. Detection level weighting

A fundamental issue of an uncertain OM determination in XFEL data in particular is that the partiality correction is systematically inaccurate. If no bias is introduced in the partiality correction estimation, it is expected that the average partiality correction will still be accurate. However, for weak reflections a bias is introduced as the peak detection limitation prohibits the integration of weak reflections if their partiality is low. In essence, a low partiality weak reflection will be hidden in the noise, whereas a strong reflection of the same partiality will be detected. Consequently, only progressively higher partialities will be detected and integrated for progressively weaker peaks. The uncertainty in the partiality correction will, in these cases, primarily result in a value lower than the true one. The effect is that after application of the partiality correction, the weak reflections are systematically overestimated. This is consistent with experimental observations and simple simulations. The experimental observations are binned based on the SNR, measured as the integrated intensity divided by the estimated noise of the frame, which serves as a rejection criterion for too-low peak intensity. The simulation is based on randomly sampling a 1D Gaussian distribution with a variable threshold intensity required to detect a peak and calculating the mean partiality of all detected peaks as the ratio of the detected intensity to the maximum intensity.

**Figure 10:**
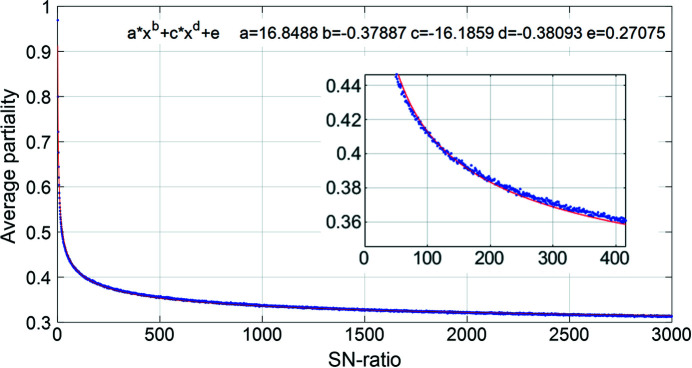
Simulated average partiality as a function of SNR. Simulated binned data points shown in blue. Each bin contains 10^6^ data points. A decent fit (red line) is obtained with the power law equation shown in the top of the plot. As shown in the inset, the fit is not flawless, but as the correction of the partiality correction is only meant to speed up the post-refinement process, the fit does not need to be perfect.

In order to eliminate the bias, a correction of the partiality correction is being implemented in the code. The systematic effect of unavoidable uncertainties can be partially circumvented prior to the post-refinement and merging of symmetry equivalent reflections by scaling reflections (binned by SNR) to their expected partiality correction as described by the fit shown in Fig. 10 ▸. This has the effect that individual reflections do not have the exact partiality predicted by the corresponding orientation matrix, which is obviously problematic. However, the OM is optimized against the expected symmetry equivalent intensity during post-refinement, meaning that the detection level weighting only affects the starting point of the post-refinement procedure. The detection level weighting approaches unity as the post-refinement converges, so all final reflection partialities are determined by refined OMs. In principle, this implementation should reach the same minimum during post-refinement, but at a faster pace. However, we find that the post-refinement results are not identical, likely owing to convergence issues in the post-refinement procedures, which are still under active development.

The current implementation is to base the correction on the binned SNR, but for homogenous particle sizes and known approximate crystal structures, it could also be based on calculated intensities or from the average intensity of families of reflections based on the initial poor partiality. The accuracy of the detection level weighting is not as important as the other corrections, as it only serves to improve convergence in post-refinement.

### 4.4. Detector gain

The uncertainty in intensity of integrated reflections can be estimated from Poisson statistics, provided that the measured intensity is on the scale of individual interactions, *i.e.* the detector signal corresponds to a photon count. CCD detectors do not inherently measure the photon flux on an absolute scale, but for the specific detector system, a conversion can be determined based on the detector gain, *G*, following the expression

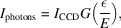

where ε is the energy necessary to generate an electron–hole pair in silicon, 3.65 [eV/e^−^], and *E* is the X-ray energy. The standard deviation can be calculated assuming Poisson statistics of the pixel intensities and the absence of correlation between pixels as (Bolotovsky *et al.*, 1995 ▸)

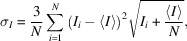

where *N* is the number of pixels contributing to the total intensity of a reflection. Counting statistics are not the sole contribution to variation; thus, σ_*I*_ should be scaled by the frame noise:

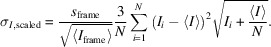




### 4.5. Hidden tails

**Figure 11:**
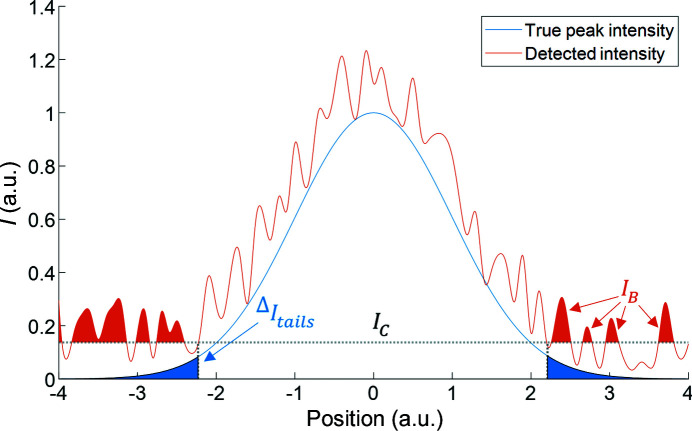
Schematic overview of the origins of the seed-skewness corrections ‘hidden tails’ and ‘included noise’. The most intense parts of the background, *I*_B_, and the weakest parts of the peak tail, Δ*I*_tails_, will be incorrectly assigned to the peak and the background, respectively.

Inherent to the seed-skewness integration method is the fact that the most intense parts of the background and the weakest parts of the peak tail will be incorrectly assigned to the peak and the background, respectively. This is schematically shown for a simulated 1D Gaussian with added random noise in Fig. 11 ▸. Here, *I*_B_ is the potentially included background pixels, and Δ*I*_tails_ is the excluded weakest parts of the peak tail. It is possible to correct for these errors on the basis of a few simple assumptions. The implementation for the hidden tails correction is based on the work by Bolotovsky *et al.* (1995 ▸, 1997 ▸).

If we estimate the diffraction spot profile to be a simple 2D isotropic Gaussian, *I*(*r*):

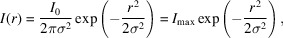

where *I*_0_ is the true integrated peak intensity, *r* is the distance from the peak center and σ is the Gaussian peak width. We can rewrite this in terms of the maximum recorded intensity, *I*_max_:

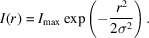

The seed-skewness implementation grows the peak area from an initial seed by adding the pixel with the highest intensity – which is adjacent to the current seed – to the seed. At some critical value, *I*_c_, the intensities of the remaining pixels become negligibly low and thus indistinguishable from the noise in the background. By rearranging the previous equation, the radius of the peak mask at this intensity can be calculated as

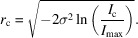

The intensity in the tails beyond the distance *r*_c_ must then be given as the integral of the intensity function from *r*_c_ to infinity:

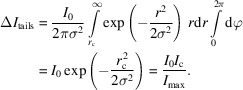

It is reasonable to expect *I*_c_ to be proportional to the background noise level with some constant of proportionality. The relative tail intensity can then be written as

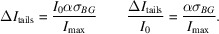

It seems intuitive that the quantity of peak intensity hidden in peak tails is inversely proportional to the SNR of the peak. The noise level is easily estimated as the root-mean-square deviation of all background pixels, *p*_BG_:

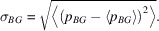

The constant of proportionality, α, can be determined by considering the statistics underlying the seed-skewness method. This has been done where the value was found to be approximately 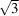 (Bolotovsky & Coppens, 1997 ▸), which corresponds well to experimental results (Bolotovsky *et al.*, 1995 ▸). The intensity corrected for the hidden peak tails can then be written as

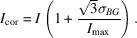




### 4.6. Included noise

At each step of seed growth, the most intense pixel adjacent to the current seed is added to the seed area. This will naturally mean that, as the peak intensity approaches the background noise level, it becomes increasingly likely that a background pixel is erroneously included. Note that although a strict boundary between peak pixels and background pixels is a necessity for the practical implementation of the seed-skewness method, in reality, accurate distinction is impossible. However, it is possible to estimate and correct for the effect on the integrated intensity of the inclusion of high-intensity background pixels. This correction for included noise is not the same as described in either of the original seed-skewness papers by Bolotovsky and Coppens (Bolotovsky *et al.*, 1995 ▸; Bolotovsky & Coppens, 1997 ▸). We found their assumption that 50% of all seed area pixels are actually background pixels to be unreasonable in most cases and instead opted to correct based on the assumption that background pixels are at the outer boundary of the peak with a computable probability. The physical background of the correction is illustrated in Fig. 11 ▸, where potentially erroneously included background pixels are shaded in orange. Note that due to our seed-skewness implementation, these pixels would only be included if adjacent to at least one peak pixel on the 2D detector frame.

The correction is based on determining the mean intensity of background pixels erroneously included in the peak and estimating the number of those pixels for each peak. We assume that background pixels are normally distributed with a density function described by a Gaussian of the shape

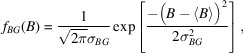

where *B* is the intensity of a background pixel. Thus, we can determine the mean intensity value of background pixels with a high enough intensity to be included in the seed area as an integral over the function

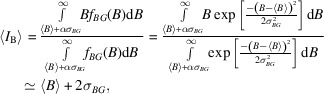

where α is 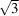 as in the hidden tails correction. The interval of the integral is based on the fact that a background pixel will be included in the seed area if the intensity is above 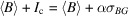 . The number of background pixels included in the seed area can be reasonably estimated based on the likelihood that an individual background pixel has an intensity exceeding 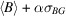 multiplied by the number of pixels at the edge of the seed area. The likelihood that an individual background pixel has an intensity exceeding 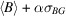 is calculated as

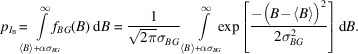

The number of pixels at the edge of the seed area, *N*_edge_, is determined directly from the peak mask used for each peak. As the mean background is already subtracted during the integration procedure, the combined correction can then be written as

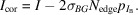




## 5. Post-refinement and merging

Prior to merging of the intensities of equivalent reflections obtained from integration, a post-refinement routine has been devised. As is standard for single-crystal diffraction, a scale is applied to each OM on each frame. The scale is determined based on the minimization of the difference of the intensity of each reflection relative to the mean intensity of equivalent reflections. As mentioned, the OM determination is inaccurate if maximization of the collective reflection partiality from a crystallite has been attempted, and it is therefore pertinent to allow a post-refinement of the OM based on corrected reflection intensities instead of spot positions. Furthermore, it is inevitable that some reflections are outliers, which need to be identified and discarded prior to final reflection merging.

Both partiality refinement and frame scale factor determination require a reasonable number of reflections to be reliable and robust. For this reason, the post-refinement is initialized with a rejection step, where reflections are rejected if the multiplicity is too low, and frames are rejected if fewer than *N*_min_ reflections are successfully integrated for a given OM.

After rejection, post-refinement proceeds iteratively. Each cycle begins with a calculation of the weighted average reflection intensity for each set of equivalent reflections. The relative weight of reflections is

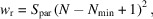

where *N* is the total number of integrated spots for the same OM. Additional spots for an OM significantly increase the accuracy of OM determination, justifying the higher weighting. Additionally, a low partiality means that the partiality is corrected by a larger factor, aggravating any errors in the reflection. The procedure prevents undue effects of outliers. The weighting scheme is being actively evaluated.

The iterative process is automatically dynamically adjusted to include additional parameters when approaching convergence. Initially, only the scale factors for individual frames are refined and new weighted averages computed. When the maximum scale factor change in a cycle is converged, a single cycle of OM refinement is included. Contrary to earlier OM refinements, it is not based on maximizing the collective partiality of reflections. Instead, the difference between corrected integrated reflection intensities and the weighted average for equivalent reflections is used as the minimization criterion in a least-squares refinement, under a restraint that guarantees consistency of the experimental and the predicted positions. The weighting of the spot position restraint is user-defined. When both the OM and the scale factor converge, a rejection step is included, removing spots with too low partiality or an intensity difference to the weighted average more than three estimated standard deviations. Subsequently the initial rejection criteria of multiplicity and spot number are reapplied, and the iterative process repeats until convergence, at which point the reflections are used to form a final structure factor list.

The pipeline is optimized heavily, with all significant calculations being parallelized and recalculation of parameters minimized. This results in computation speeds that allow the software to be used during a measurement to optimize the experiment.

## 6. Data reduction and crystal structure refinement for PtPOP

In total, 695 000 data frames were measured on a sample of PtPOP single crystals dispersed in a grease matrix (Sugahara *et al.*, 2015 ▸). However, many of the frames did not contain any reflections as no crystals were hit during their measurement. In addition, some frames contained too few reflections to initiate the indexing process, and some frames contained too many reflections, indicating that we might have hit a cluster of crystals. In order to speed up the data analysis, a preprocess was therefore performed, skimming through the data and removing any frames with an absolute intensity or a relative intensity compared with the mean below our user-defined thresholds (50 000 and 1000, respectively) as well as any frames containing fewer than 5 or more than 100 spots. After running the peak hunting process of the pipeline, ∼50 400 frames remained for the indexing process, corresponding to ∼7.3% of the total number of collected frames. Initially, the total number of identified reflections on the frames was ∼477 500, giving an average of 9–10 spots per frame. Some of the reflections were flagged because they were bleeding, some because they were on the border of the detector panels, some because they had non-ideal intensity distributions *etc*. After the initial indexing process, ∼19 100 frames remained, and approximately 65% of the initially found reflections were indexed, corresponding to ∼312 200 peaks. During the integration process, ∼149 000 reflections were successfully integrated. Around 50 frames were rejected during this integration, resulting in an average number of 8 integrated reflections per frame. During the post-refinement and scaling process, ∼56 400 reflections were rejected and approximately half of the frames were discarded. In the end, after around 80 refinement cycles, approximately 92 600 reflections from ∼9800 frames were written to the final *hkl* file needed for the subsequent structure-solving process. This corresponds to an average of 9–10 reflections per frame. Note that the final reflections are not necessarily in the initial pool of reflections as additional reflections can be included at later stages of the post-refinement. The final *R*_int_ value was 0.258, and the completeness was 81% up to a *d* spacing of 0.84 Å. The statistics for the data analysis of the PtPOP data using the pipeline are summarized in Table 2 ▸.

**Figure 12:**
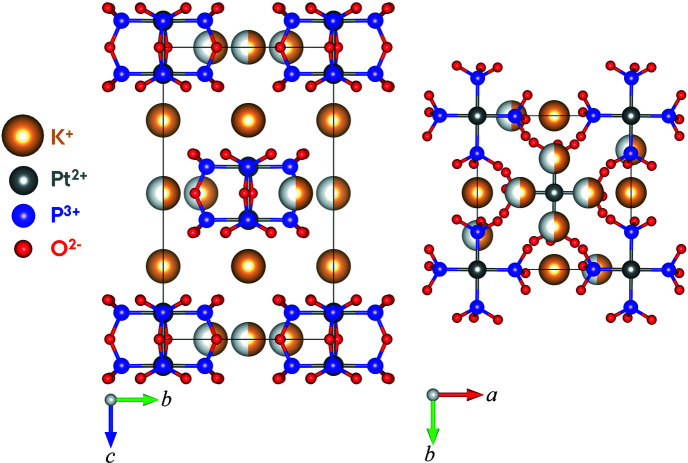
*I*4/*m* structure of K_4_[Pt_2_(P_2_O_5_H_2_)_4_]·2H_2_O determined from the SACLA data. Water molecules and hydrogens have been omitted.

For the PtPOP data obtained at SACLA, the structure-solving process was based on the *hkl* file produced by the data reduction pipeline as well as the unit-cell parameters of the PtPOP structure determined at our in-house single-crystal diffractometer (SuperNova). The structure-solving process was performed in *Olex2* with *SHELXS* (Sheldrick, 2008 ▸) using the Patterson method, and a subsequent refinement was executed with *SHELXL* (Sheldrick, 2015*b* ▸) using a weighting scheme and ADPs. Furthermore, an extinction correction was included to correct for a, thus far, unresolved systematic deviation of the calculated structure factors from the observed ones. The refinement details are summarized in Table 3 ▸, and the structure obtained for K_4_[Pt_2_(P_2_O_5_H_2_)_4_]·2H_2_O is shown in Fig. 12 ▸. The final *R*_1_ value was ∼9.1%. Note that crystalline water molecules and hydrogens have been omitted from the structure presented in Table 3 ▸ and Fig. 12 ▸.

The structure of K_4_[Pt_2_(P_2_O_5_H_2_)_4_]·2H_2_O determined from the data measured at SACLA visualized in Fig. 12 ▸ can be compared to the reference structure obtained from the SuperNova data shown in Fig. 1 ▸. Note that the axes on the two figures differ, corresponding to a rotation of 180° of one of the unit cells around the *b* axis compared with the other cell. However, as the ‘top’ and ‘bottom’ of the unit cell are arbitrarily defined, this is insignificant for the comparison of the two structures. By comparing Fig. 12 ▸ with Fig. 1 ▸, it is evident that the PtPOP structure obtained from the data collected at SACLA and processed in our pipeline is ultimately the same structure as the one determined from the data measured with the SuperNova diffractometer. The resemblance between the two structures is further supported by the similarities in bond distances and angles, of which a selection are displayed in Table 4 ▸. Typically, the bond lengths differ on the order of 0.01 Å. Considering that structural changes in excited electronic states can easily reach 0.1 Å, we conclude that the present data reduction pipeline appears to provide a data quality suitable for pump–probe serial crystallography on small-unit-cell structures.

## 7. Conclusions

XFEL small-unit-cell crystallography has the potential to allow exciting new studies into dynamic properties of relevant materials without difficult sample requirements. Until now, however, this has been a largely unexplored research area due to the difficulties of data reduction of serial crystallography with a low number of diffraction spots. Here, we present a robust and flexible method for automatic data reduction featuring accurate integration and data correction in a heavily optimized computational framework based on the knowledge of the unit-cell parameters and the crystal Laue class. The pipeline writes intermediate results between the spot finding, indexing, integration and final merging of the structure factor list, allowing easy integration with other data treatment methods.

An initial proof of the quality of both data and reduction lies in the quality of structure solution and refinement. Therefore, serial femtosecond single-crystal X-ray diffraction data measured on the small-unit-cell system K_4_[Pt_2_(P_2_O_5_H_2_)_4_]·2H_2_O have been analyzed. Using our data reduction pipeline, we managed to find, index and integrate spots on data frames with a very sparse number of reflections. Our results show a high accuracy and no systematic deviation in orientation matrix alignment for frames with as few as five diffraction spots. After applying the vast number of intensity corrections and running the data through the post-refinement process, the final structure factor list obtained from the pipeline allowed for a successful structural determination. The obtained accuracy of the structure solution and refinement (*R*_1_ ≃ 9.1%) is inferior to a laboratory-based crystal structure analysis on the same crystals (*R*_1_ ≃ 3.7%), but a comparison reveal that the refined bond length typically differs only on the order of 0.01 Å. This gives promise that structural changes, for example during laser excitation, can be resolved. Optimization of the structure solution is primarily a matter of improving the partiality correction, which is likely to be the main source of error as it is a direct multiplier of all other errors accumulated in a reflection intensity.

## Supplementary Material

Crystal structure: contains datablock(s) SACLA. DOI: 10.1107/S2052252522011782/it5028sup1.cif

Crystal structure: contains datablock(s) SuperNova. DOI: 10.1107/S2052252522011782/it5028sup2.cif

CCDC references: 2225399, 2226939

## Figures and Tables

**Table 1:** Experimental details for K_4_[Pt_2_(P_2_O_5_H_2_)_4_]·2H_2_O Hydrogen atoms and water molecules have been omitted.

Crystal data	
Chemical formula	K_4_Pt_2_P_8_O_20_
*M*_r_	1158.44
Crystal system, space group	Tetragonal, *I*4/*m*
Temperature (K)	293
*a*, *c* (Å)	9.3113 (3), 15.9547 (11)
*V* (Å^3^)	1383.28 (13)
*Z*	2
Radiation type	Mo *K*α
μ (mm^−1^)	11.25
Crystal size (mm)	0.046 (radius)

Data collection	
Diffractometer	SuperNova, Single Source at Offset/far, Atlas
Absorption correction	Multiscan absorption correction as implemented in CrysAlisPro
*T*_min_, *T*_max_	0.468, 0.480
No. of measured, independent and observed [*I* > 2σ(*I*)] reflections	7706, 1074, 1023
*R*_int_	0.048
(sinθ/λ)_max_ (Å^−1^)	0.724

Refinement	
*R*[*F* ^2^ > 2σ(*F* ^2^)], *wR*(*F* ^2^), *S*	0.037, 0.106, 1.16
No. of reflections	1074
No. of parameters	45
	*w* = 1/[σ^2^(*F*_O_^2^) + (0.0573*P*)^2^ + 22.3142*P*] where *P* = (*F*_O_^2^ + 2*F*_C_^2^)/3
Δρ_max_, Δρ_min_ (eÅ^−3^)	4.33, −1.03

Computer programs: *CrysAlis PRO* (RigakuOD, 2021 ▸), *SHELXT* (Sheldrick, 2015*a* ▸), *SHELXL* (Sheldrick, 2015*b* ▸), *Olex2* (Dolomanov *et al.*, 2009 ▸).

**Table 2:** Pipeline statistics for the data reduction of the PtPOP data measured at SACLA Note: the final reflections are not necessarily in the initial pool of reflections.

	No. of frames	No. of reflections
Initial	∼695000	–
After peak-hunting	∼50400	∼477500
After indexing	∼19100	∼312200
After integration	∼19000	∼149000
After post-refinement and scaling	∼9800	∼92600

**Table 3:** Experimental details for the PtPOP data measured at SACLA Note that crystalline water molecules and hydrogens have been omitted.

Data collection	
Diffractometer	Bruker APEX-II CCD
No. of measured, independent and observed [*I* > 2σ(*I*)] reflections	92219, 526, 526
*R*_int_	0.265
(sinθ/λ)_max_ (Å^−1^)	0.615

Refinement	
*R*[*F* ^2^ > 2σ(*F* ^2^)], *wR*(*F* ^2^), *S*	0.091, 0.235, 1.20
No. of reflections	526
No. of parameters	46
	*w* = 1/[σ^2^(*F*_O_^2^) + (0.1802*P*)^2^ + 43.3637*P*] where *P* = (*F*_O_^2^ + 2*F*_C_^2^)/3
Δρ_max_, Δρ_min_ (eÅ^−3^)	3.83, −2.11

Computer programs: *xprep* (Bruker, 2014 ▸), *SHELXS* (Sheldrick, 2008 ▸), *SHELXL* (Sheldrick, 2015*b* ▸), *Olex2* (Dolomanov *et al.*, 2009 ▸).

**Table 4:** Selected bond distances (*d*) and angles (θ) in the K_4_[Pt_2_(P_2_O_5_H_2_)_4_]·2H_2_O structure obtained from data measured on an in-house single-crystal diffractometer (SuperNova) and at SACLA

	SuperNova	SACLA
*d*(Pt—Pt) (Å)	2.8939 (7)	2.886 (6)
*d*(Pt—P) (Å)	2.3133 (16)	2.279 (13)
*d*(P=O) (Å)	1.517 (5)	1.52 (4)
*d*(P—OH) (Å)	1.582 (5)	1.63 (4)
*d*(—O—K) (Å)†	2.776 (5)	2.75 (3)
*d*(=O—K) (Å)†	2.819 (5)	2.83 (4)
θ(P—O—P) (°)	130.9 (5)	131 (4)

† The two distances between O and K are for the K^+^ ions at the 4*d* sites and their nearest and next-nearest oxygen neighbors, respectively.
